# Superb microvascular imaging (SMI) compared with conventional ultrasound for evaluating thyroid nodules

**DOI:** 10.1186/s12880-017-0241-5

**Published:** 2017-12-28

**Authors:** Ruigang Lu, Yuxin Meng, Yan Zhang, Wei Zhao, Xun Wang, Mulan Jin, Ruijun Guo

**Affiliations:** 1 Department of Ultrasonography, Beijing Chao-Yang Hospital, Capital Medical University, Beijing, 100020 China; 2 Department of Endocrinology, Beijing No. 6 Hospital, Beijing, 100007 China; 3 Department of Otorhinolaryngology Head and Neck Surgery, Beijing Chao-Yang Hospital, Capital Medical University, Beijing, 100020 China; 4 Department of Pathology, Beijing Chao-Yang Hospital, Capital Medical University, Beijing, 100020 China

**Keywords:** Superb microvascular imaging, Thyroid nodule, CEUS, Microvascular, CDI/PDI, Microvessel density

## Abstract

**Background:**

Superb microvascular imaging (SMI) for depiction of microvascular flow in thyroid nodules was compared with color/power Doppler imaging (CDI/PDI) and contrast-enhanced ultrasonography (CEUS). In addition, the diagnostic performance of conventional ultrasound combined with SMI for differentiating benign and malignant thyroid nodules was evaluated.

**Methods:**

Preoperative conventional ultrasound consisting of gray-scale ultrasonography and CDI/PDI, followed by SMI and CEUS, was used to record 52 thyroid nodules. Two radiologists analyzed the gray-scale ultrasound signs and nodules’ microvascular flow patterns to differentiate between benign (*n* = 13) and malignant nodules (*n* = 39).

**Results:**

SMI was significantly more effective in the detection of microvascular flow signals than CDI/PDI. In malignant nodules, SMI depicted the presence of incomplete surrounding periphery microvasculature and of disordered heterogeneous internal microvasculature. Benign nodules showed complete surrounding periphery microvasculature (ring sign) and homogeneity internal branching. The accuracies of conventional ultrasound combined with CDI/ PDI, SMI, or CEUS for predicting malignancy were 67.31, 86.54, and 92.31%, respectively. The accuracy of SMI differed significantly from CDI/PDI (*P* = 0.012), but not from CEUS (*P* = 0.339).

**Conclusions:**

Microvascular flow and vessel branching in the peripheral and internal microvasculature of thyroid nodules is depicted with greater detail and clarity with SMI compared with conventional ultrasound. SMI offers a safe and low-cost alternative to CEUS for differentiating between benign and malignant thyroid nodules.

## Background

Thyroid nodules are a common finding. With development of ultrasonic technology and improvement in resolution, the rate of detection of thyroid nodules has risen, especially in younger persons [1]. In clinical practice, ultrasound is the primary imaging method for evaluating thyroid nodules. However, some of the typical characteristics of malignant nodules have a diagnostic accuracy of only 74 to 82%, including hypoechogenicity, irregular margins, being taller than wide, microcalcifications, absence of halo, and presence of perforator vessel(s) [2, 3]. None of these features is specific enough to classify a lesion as malignant, and sensitivity is also significantly lower for any single feature [4]. It is therefore important to explore a complementary method which could improve the identification of benign and malignant nodules.

It is well known that blood vessels are important to the growth of a nodule, but conventional color or power Doppler imaging (CDI and PDI, respectively) are not very sensitive to microvascularity patterns and low blood flow velocity [5]. Vessel extraction methods, such as minimal path techniques to effectively locate tiny vessels, might be of help to extract vessel-related information for further diagnosis [6], although they are limited by some inherent problems like endpoint, shortcut and accumulation issues [7]. A recent improved means of visualizing low flow in microvessels is superb microvascular imaging (SMI) ultrasound technology, implemented with the Aplio 500 US system by Toshiba (Toshiba Medical Systems, Tokyo, Japan). SMI uses advanced clutter suppression to extract flow signals from large to small vessels, and depicts this information at high frame rates as a color overlay image or as a grayscale map of flow (color or monochrome SMI, respectively).

The present study compares the flow imaging abilities of SMI, CDI/PDI, and CEUS for depicting microvascular flow in thyroid nodules, and evaluates the efficacy of these methods for identifying benign and malignant thyroid nodules.

## Methods

The study was conducted from March to November 2016. Fifty prospective patients (45 women and 5 men; 52 tumors) who were referred for surgery due to suspicion of malignancy after gray-scale ultrasound or palpation, were enrolled. The mean age of these patients was 47 years (range: 20-65 years).

All patients enrolled underwent ultrasound examination of the thyroid consisting of the following modalities: gray-scale ultrasound; CDI; PDI; color and monochrome SMI; and CEUS using an Aplio 500 ultrasound system with a broad bandwidth linear array transducer (imaging frequency, 14 MHz). The nodules studied were solid or cystic solid with a solid component greater than 50% and a maximum diameter of 0.5-3.0 cm. For all subjects, the settings for cross section, constant color sampling frame size, and moderation scale were identical throughout the imaging portion of the study. In selected patients, pulsed Doppler was also used during monochrome SMI to examine a few specific microvessels inside the nodule, to verify that the observed vessels were real microvasculature.

Two senior radiologists read all the fixed and dynamic pictures independently. Nodules were scored by analyzing microvasculature and branching using a visual-analog scale of 0 (worst) to 10 (best), based on the semi-quantitative percentage of nodule volume occupied by vessel signals. For each patient, the following characteristics were recorded: vessel branching detail, peripheral vascularization surrounding the nodule, and homogeneous or heterogeneous disordered distribution of internal small branching microvasculature.

Images and cine loops of the thyroid nodules from all 5 modalities, acquired during the ultrasound examinations, were independently analyzed by 2 radiologists (blinded to the histology pathology results). The observers independently scored the digital clips from each of the 5 flow modes, and each nodule with regard to overall flow detection and vessel branching details, scoring them as 0 (worst) to 10 (best). The qualitative scores (i.e., for overall flow detection and vessel branching details) were compared on a per nodule basis using a nonparametric Wilcoxon signed rank test.

All tests were performed using Stata 12.0 software (Stata Corp, College Station, TX), with *P* < 0.05 indicating statistical significance. To assess the consistency of the vascularity scores determined by the 2 observers, k-coefficients were calculated.

## Results

**Fig. 1:**
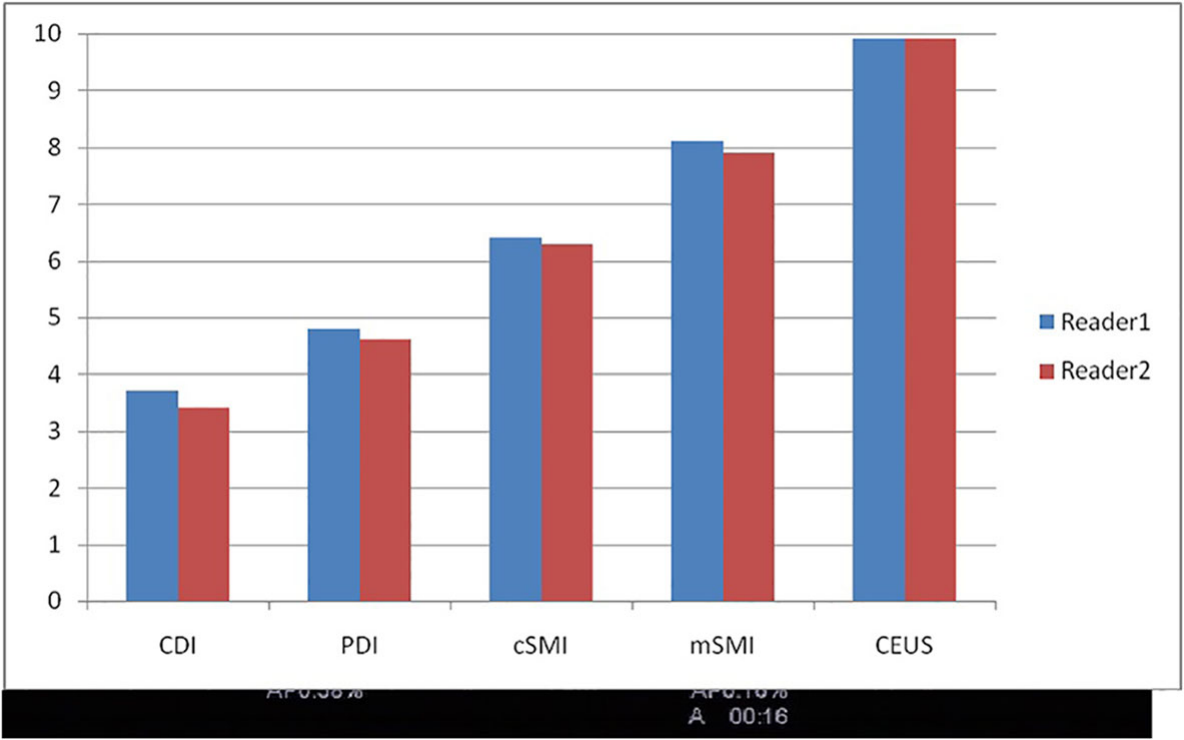
Overall flow detection scored on a subjective scale of 0 to 10 for CDI, PDI, color SMI (cSMI), monochrome SMI (mSMI), and CEUS of average distribution for all nodules

For the 52 solid nodules, the results of histology yielded 39 malignant (papillary thyroid carcinoma) and 13 benign nodules. The latter consisted of 5 follicular adenomas, 6 nodular goiters, 1 parathyroid adenoma, and 1 shrinking cyst. The details of microvasculature within the nodules were recorded semi-quantitatively (Fig. 1).

**Fig. 2:**
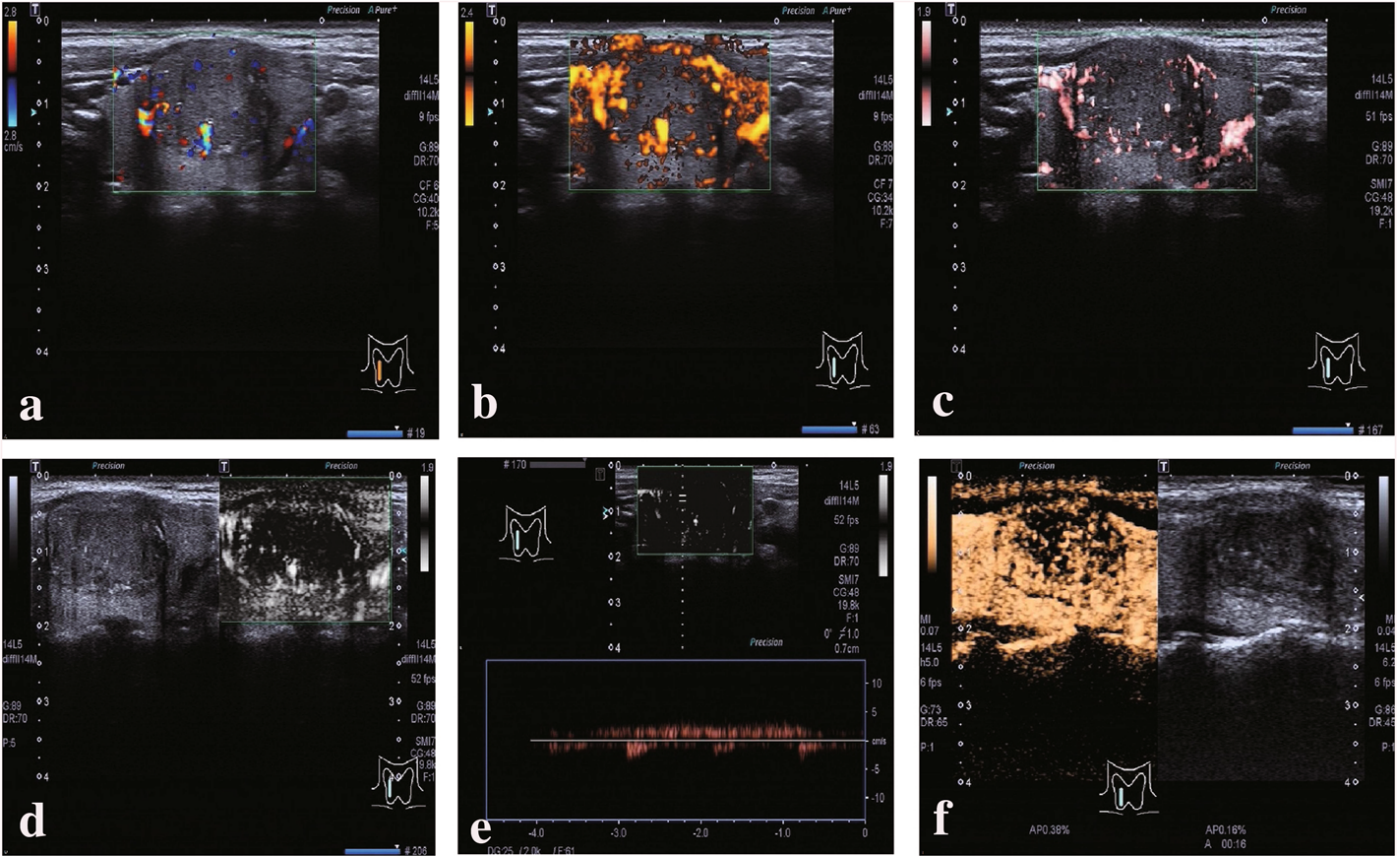
A papillary thyroid carcinoma located in the right lobe. **a** CDI and **b** PDI show peripheral and internal vascularity of the nodule, with **c** color SMI and **d** monochrome SMI depicting more detailed peripheral vascularity surrounding the nodule as well as internal disordered small branching microvessels. **e** Pulsed Doppler (guided by monochrome SMI) established that the small branching microvasculature was real and measurable. **f** CEUS features of the nodule. The nodule enhances later compared with normal thyroid parenchyma, with disordered heterogeneous hypoechoic enhancement and incomplete peripheral enhancement

**Fig. 3:**
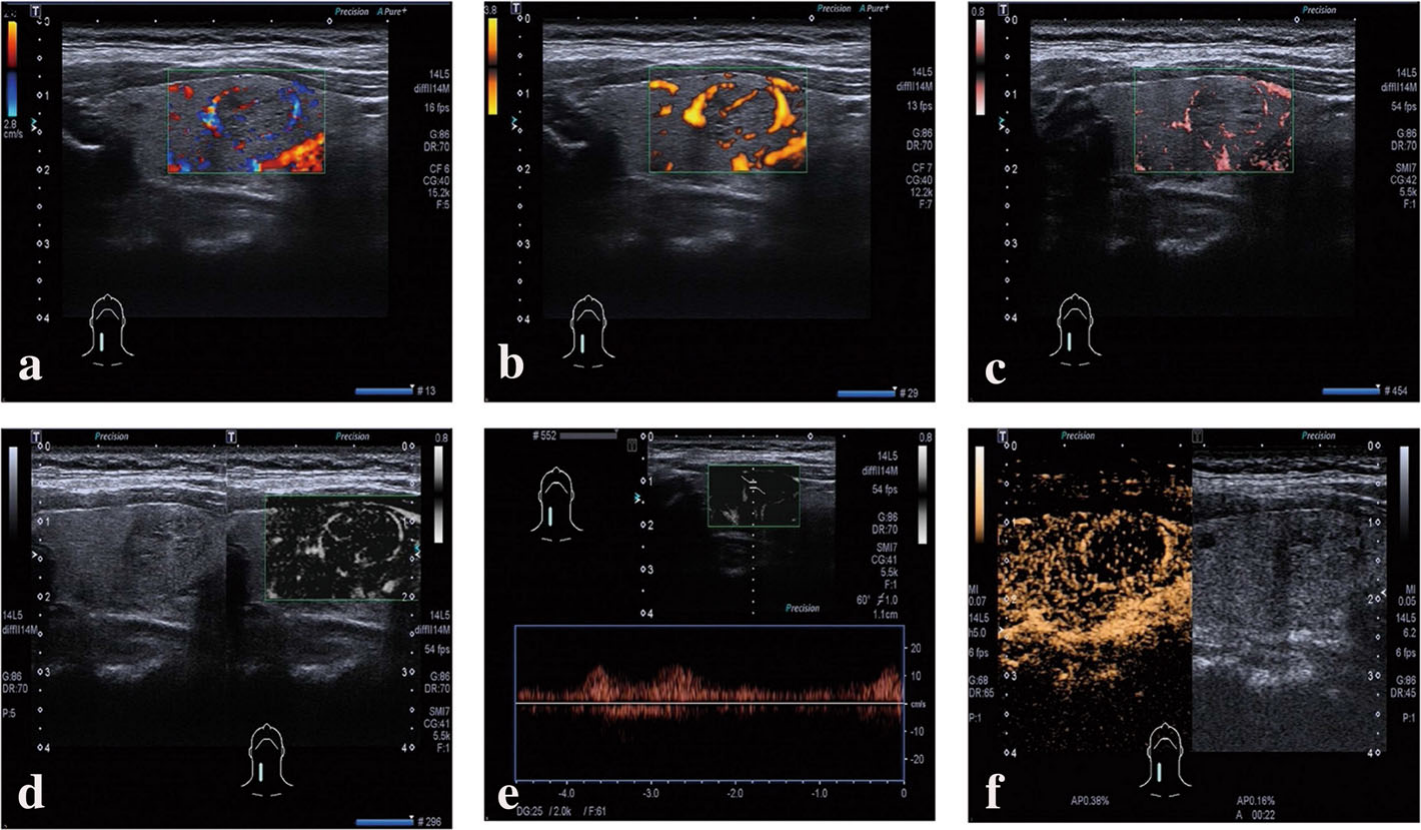
An isoechoic thyroid nodule located in the right lobe. **a** CDI and **b** PDI show some peripheral vascularization around the nodule. **c** Color SMI and **d** monochrome SMI show complete peripheral vascularization surrounding the nodule, with internal microvascularization and small branching details. **e** Pulsed Doppler (guided by monochrome SMI) established that the small branching microvasculature was real and measurable. **f** CEUS shows ring enhancement around the nodule and homogeneous internal enhancement. Histology: adenoma

**Fig. 4:**
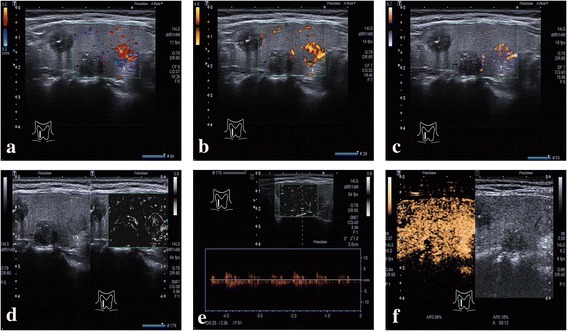
A papillary thyroid carcinoma (➔) and an adenoma (⇑) coexist in the right lobe. **a** CDI and **b** PDI show peripheral vascularization and peripheral interrupted vascularization of the nodule and internal microvascularization. **c** Color SMI and **d** monochrome SMI show more vascularization, in particular curved and ring peripheral vascularization surrounding the nodule as well as internal microvascularization with small branching details. **e** Pulsed Doppler (guided by monochrome SMI) established that the small branching microvasculature was real and measurable. **f** CEUS shows disordered heterogeneous hypoechoic enhancement with incomplete peripheral enhancement (➔) and ring enhancement around the nodule and homogeneous hyperechoic internal enhancement (⇑).

Both color and monochrome SMI delivered clearer and more cohesive vessel branching detail in the nodules compared with CDI/PDI. In malignant nodules, SMI revealed disordered heterogeneous internal microvessel distribution, scarce vascularity in hypoechoic nodules or interrupted surrounding peripheral microvasculature (Figs. 2 and 4). In benign nodules, SMI showed complete surrounding periphery microvasculature and homogeneous internal microvessel distributions (Figs. 3 and 4). The characteristics of the nodules according to microvascular flow of CDI/PDI, SMI, and CEUS were evaluated and the accuracy of the 3 imaging microvascular flow patterns was assessed.

Pulsed Doppler guided by monochrome SMI established that the small branching microvessels detected by monochrome SMI are real and can be measured (Figs. 2e, 3e and 4e). CEUS features of the nodule (Figs. 2f, 3f and 4f). Observations included heterogeneous hypoechoic enhancement of the middle nodule, and homogeneous hyperechoic enhancement in the right nodule (Figs. 4f). The results for the various ultrasound modalities in the depiction of microvascular flow in thyroid nodules are summarized in Table 1.

**Table 1:** CDI/PDI, SMI, and CEUS for depiction of microvascular flow distribution in thyroid nodules

	Disordered heterogeneous	Homogeneous	Ring	None	Sum
CDI/PDI	40	3	3	6	52
SMI	38	5	7	2	52
CEUS	37	6	8	1	52

The consistency of scores for a subset of 52 nodules among the CDI/PDI, SMI, and CEUS images acquired by 2 independent operators was assessed. The k-coefficients of CDI/PDI, SMI, and CEUS were 0.949, 0.871, and 0.918, respectively. The diagnostic accuracies of the 3 methods are presented in Table 2. The accuracies of conventional ultrasound combined with CDI/ PDI, SMI, or CEUS for predicting malignancy were 67.31, 86.54, and 92.31%, respectively. SMI differed significantly from CDI/PDI (χ2 = 6.372, *P* = 0.012), but not from CEUS (χ2 = 0.915, *P* = 0.339).

**Table 2:** Diagnostic accuracy of conventional ultrasound combined with CDI/PDI, SMI and CEUS in predicting benign and malignant thyroid nodules

	Benign or malignant		
	Conform	Not conform	Sum
CDI/PDI	35	17	52
SMI	45	7	52
CEUS	48	4	52

## Discussion

Ultrasound is the primary imaging method for the evaluation of thyroid nodules [4]. Flow imaging of thyroid nodules can differentiate between benign and malignant nodules. However, there are technical limitations in using CDI/PDI for detecting small blood vessels and low blood flow. Currently, microvessel density is the gold standard for determining tumor angiogenesis [8, 9]. The radiological evaluation of tumor angiogenesis includes morphological abnormalities of the microvessels, and differences in neonatal microvascular density and function [10, 11]. However, microvessel density can only be measured postoperatively, and therefore cannot be used to prevent unneeded invasive procedures, that is, for identifying benign lesions that should not be operated on.

SMI is a relatively new imaging modality that is implemented on the Toshiba Aplio 500 ultrasound system. It enables better depiction of microvascularity and low-velocity blood flow compared with conventional ultrasound methods. Low-level echoes that are near the noise level of the equipment, are detected by the SMI procedure for maximum information with minimum noise, but improper adjustment of the system gain may cause SMI imaging information distortion. In the present study, SMI did indeed reveal more small branches of microvasculature compared with CDI/PDI, and show the distribution inside nodules and adjacent thyroid parenchyma in better detail. Judged by the detail in its images, SMI is highly accurate.

According to several reports, CEUS is able to depict microperfusion in thyroid nodules. Zhang et al. [3] demonstrated that ring enhancement was closely associated with the benign character of lesions, whereas heterogeneous enhancement was helpful for detecting malignant lesions. Jiang reported that lower contrast enhancement levels are an important diagnostic indication of tiny papillary thyroid carcinoma [12]. This study compared SMI with CEUS and found that SMI provides better display of the network of small microvascular branches and their distribution in nodules, although CEUS blood pool imaging can show thicker blood branches and vascular perfusion clearly. CEUS is believed by some researchers to have a high sensitivity to blood flow, resulting in coverage of real blood vessels [13]. However, another characteristic of CEUS is harmonic imaging, coupled with low mechanical index, low frequency, and a low frame rate (6-13 fps), which unfortunately affects the 2-dimensional display of the image. Another drawback of CEUS is variation in the contrast agent in-out time and patients’ swallowing, resulting in an unsatisfactory 3-dimensional display of microvasculature.

In the present study, SMI and CEUS were similar in the high consistency of differentiation between benign and malignant nodules. The results of conventional ultrasound combined with CEUS tended to be better than those combined with SMI, but SMI is also the more economical method and avoids the hazards associated with the contrast agent used in CEUS. Furthermore, SMI has already shown potential in the characterization of brain tumors prior to surgery [14] and in the early diagnosis of breast cancer [15, 16]. Zhan et al. [14] considered the value of SMI when combined with conventional ultrasound without contrast administration and determined that SMI was helpful in the differential diagnosis of benign and malignant avascular breast lesions, especially those in BI-RADS category 4.

To the best of our knowledge there is only one previous comparison in the literature of the diagnostic value of SMI and CEUS for the differentiation of tumors. This is a retrospective study by Xiao et al., [16] who compared the diagnostic utility of SMI and CEUS for breast lesions, using root hair-like and crab claw-like patterns as criteria for malignant lesions. Based on the microvascular architecture patterns, the sensitivity, specificity, and accuracy of monochromatic SMI for differentiation were 78, 91, and 85%, respectively, and the corresponding rates using CEUS were 90, 88, and 89%. Areas under the curve for monochromatic SMI and CEUS were not significantly different (*P* = 0.013) [15]. These breast cancer data are closely similar to the thyroid cancer data obtained in the present prospective study comparing SMI and CEUS for differentiating malignant from benign thyroid nodules. That is, we report an ~87% accuracy for SMI combined with conventional ultrasound, and a ~92% accuracy for CEUS with conventional ultrasound (*P* = 0.339). The main limitation in this study, is formed by the homogeneous background of the patients with none of the nodules being follicular carcinoma. Those typically require hemi-thyroidectomy or lobectomy for definitive diagnosis.

Our results indicate that SMI has the potential to become the method of choice for detecting microvasculature in thyroid nodules. This conclusion is in accord with previous studies showing the use of SMI for better characterization of the vascularity of breast tumors [14, 15]. Blood flow detection and depiction of microvessel architecture are superior with this new noninvasive ultrasound imaging method. SMI combined with conventional ultrasound may become the tool of choice for differentiating benign from malignant thyroid nodules. Furthermore, because SMI does not require a contrast agent, it is more economical than CEUS and avoids the adverse reactions associated with the contrast agent.

## Conclusion

SMI detects more small branches of microvasculature compared with CDI/PDI, and also shows the distribution inside nodules and adjacent thyroid parenchyma in better detail. SMI and CEUS are similar in the high consistency of differentiation between benign and malignant nodules, but SMI is also the more economical method and avoids the hazards associated with the contrast agent used in CEUS. Compared with conventional ultrasound, SMI therefore offers a superior alternative for the differentiation of benign and malignant thyroid nodules.

## Abbreviations

CDI/PDI: Color/Power Doppler imaging
CEUS: Contrast-enhanced ultrasonography
SMI: Superb microvascular imaging
